# Effect of fast‐food environments on children's eating behaviour: A random effect within between analysis within the Generation R Study

**DOI:** 10.1111/ijpo.13175

**Published:** 2024-09-29

**Authors:** Thera A. M. Peeters, Famke J. M. Mölenberg, Pauline W. Jansen, Joost Oude Groeniger, Frank J. van Lenthe, Mariëlle A. Beenackers

**Affiliations:** ^1^ Department of Public Health Erasmus MC Rotterdam The Netherlands; ^2^ The Generation R Study Group Erasmus MC Rotterdam The Netherlands; ^3^ Department of Child and Adolescent Psychiatry/Psychology Erasmus MC Rotterdam The Netherlands; ^4^ Department of Psychology, Education and Child Studies Erasmus University Rotterdam The Netherlands; ^5^ Department of Public Administration and Sociology Erasmus University Rotterdam The Netherlands; ^6^ Department of Human Geography and Spatial Planning Utrecht University Utrecht The Netherlands

**Keywords:** children, eating behaviour, food environment, longitudinal, parenting, within‐between effects

## Abstract

**Background:**

Focussing on appetitive traits associated with obesity, this study aimed to estimate the association between the fast‐food environment and satiety responsiveness, enjoyment of food and food responsiveness.

**Methods:**

We used data from the Generation R Study. We included 2008 children with repeated measurements at the age of 4–10 years old. Three eating behaviour subscales from the Child Eating Behaviour Questionnaire (CEBQ) were used as outcomes. Geographical Information System data were used to map individual‐level exposure to fast‐food outlets within 400 m from home. Random Effect Within Between (REWB) models were used to derive estimates. We tested for moderation of the associations with parental restriction at baseline using the parent‐reported Child Feeding Questionnaire (CFQ).

**Results:**

We did not find evidence of between‐associations of fast‐food exposure and eating behaviour subscales. Considering within‐associations, an increase in absolute fast‐food exposure was associated with a significant marginal increase in satiety responsiveness (*β*: 0.02 [95% confidence interval: 0.00–0.03]). No moderation by parental restriction was found.

**Conclusions:**

In environments with ubiquitous fast‐food outlets, an increased exposure to fast‐food outlets does not seem to have a substantial impact on eating behaviour. Further research is needed to better understand how fast‐food exposure contributes to overweight.

**Abbreviations**
CEBQ: Child Eating Behaviour Questionnaire
CFQ: Child Feeding Questionnaire
MIS: mean item score
REWB: Random Effect Within Between
SD: standard deviation

## 1 BACKGROUND

Childhood overweight and obesity are a leading public health concern considering their chronic character, the associated disease burden and the global increase of childhood obesity prevalence.^1^ The obesity epidemic should be understood as a complex issue caused by the interaction between the environment and the individual, whereby the food environment plays a role in shaping obesogenic behaviours.^2^, ^3^, ^4^ Over the last decade, the built retail food environment has changed considerably, offering more unhealthy ready‐to‐eat and convenience foods and promoting increased energy consumption.^1^, ^5^ Today's unhealthy food environments place the population at increasing risk by exploiting people's biological, psychological, social and economic vulnerabilities, undermining people's abilities to make healthy choices.^2^ Previous research, of which the majority were cross‐sectional in design, found inconclusive evidence on the effect of the fast‐food environment on childhood overweight and obesity.^6^, ^7^, ^8^, ^9^, ^10^ Notably, obesity‐related outcome measures are relatively distal to the exposure,^11^ and environmental changes may occur slowly and not result in changes in distal outcome measures during the observed follow‐up time.^12^ Focussing on more proximate outcomes might illuminate the pathway through which the food environment can impact people.

Eating behaviours, which reflect differences in appetitive traits, have been associated with overeating and obesity.^13^, ^14^, ^15^ The behavioural susceptibility theory states that the interplay between genetic susceptibility and exposure to an obesogenic food environment leads to overeating and adiposity. Appetite hereby mediates the genetic susceptibility to the obesogenic food environment and can be considered the behavioural expression of obesity risk.^14^, ^15^, ^16^ Rather than fixed personal characteristics, however, moderate correlations indicate that there is room for potential individual variation in the development of eating behaviours.^17^ Of interest is whether changing food environments might play a role in the development and expression of these eating behaviours. Boutelle et al. have used the behavioural susceptibility theory to describe how, through learning processes, the environment can influence appetite.^18^ When food cues are associated with food intake, over time, food cues can elicit food cravings, urges and motivation to eat.^18^

When studying the association between changing food environment and changing eating behaviours in children, the role of the parents cannot be ruled out. Parental feeding practices might moderate this relationship not only through their own eating behaviours (which in turn may be influenced by the food environment also), but also by controlling dietary intake of their children.^19^, ^20^, ^21^, ^22^ Parents' restrictions and limit setting towards foods is particularly interesting when considering that children need to learn to navigate the current obesogenic food environment. Experimental studies show that initial restriction led to increased intake of restricted food items at a later stage, potentially because of a pre‐occupation with the restricted foods.^23^, ^24^ Longitudinal evidence focusing more on longer term effects similarly indicated that restrictive feeding practices predicted more food approach behaviours as well as eating in the absence of hunger (a measure indicative of a poor satiety responsiveness), potentially because these feeding practices may impair children's ability to self‐regulate and respond appropriately to internal signals of hunger and satiety.^25^, ^26^ On the other hand, a few recent longitudinal studies also point at reverse effects with parents using more restrictive feeding practices in response to a child's food approach behaviours or unhealthy weight, suggesting that parents may adapt their strategies, for example, when having weight‐related worries.^27^, ^28^, ^29^ Indeed, a recent review of 44 (mostly cross‐sectional) studies concluded that instead of restriction being detrimental for children's dietary outcomes, it may be unrelated, or even associated with more beneficial dietary outcomes.^30^

Consequently, the objective of this study was to estimate to what extent changes in the expression of eating behaviours are externally motivated by changes in the fast‐food environment in school aged children and to what extend this association is moderated by parental restriction. We hypothesized that unhealthy food environments play a significant role in the increased expressions of food approach behaviours and decreased satiety responsiveness. Parental feeding practices are hypothesized to moderate this relationship, whereby we expect that more restrictive feeding practices will lead to decreased levels of self‐control, leading to increased food approach behaviours and decreased satiety responsiveness in the face of increasing fast‐food exposure.

## 2 METHODS

### 2.1 Study design and population

This study used data collected by the Generation R cohort in Rotterdam, The Netherlands, for which a more detailed description has been given previously by Kooijman et al.^31^ The Generation R Study was approved by the Medical Ethical Committee of the Erasmus University Medical Center in Rotterdam (MEC 198.782/2001/31).

Summarized, pregnant women living in Rotterdam with an expected delivery date between April 2002 and January 2006 were invited to participate in this prospective birth‐cohort. A total of 9778 mothers enrolled, with a number of 9749 live births. We included children who participated during the preschool period at the age of 4, and children of 10 years old that participated in the focus@9 research wave, considering available data on eating behaviour at these measurement waves.

**FIGURE 1:**
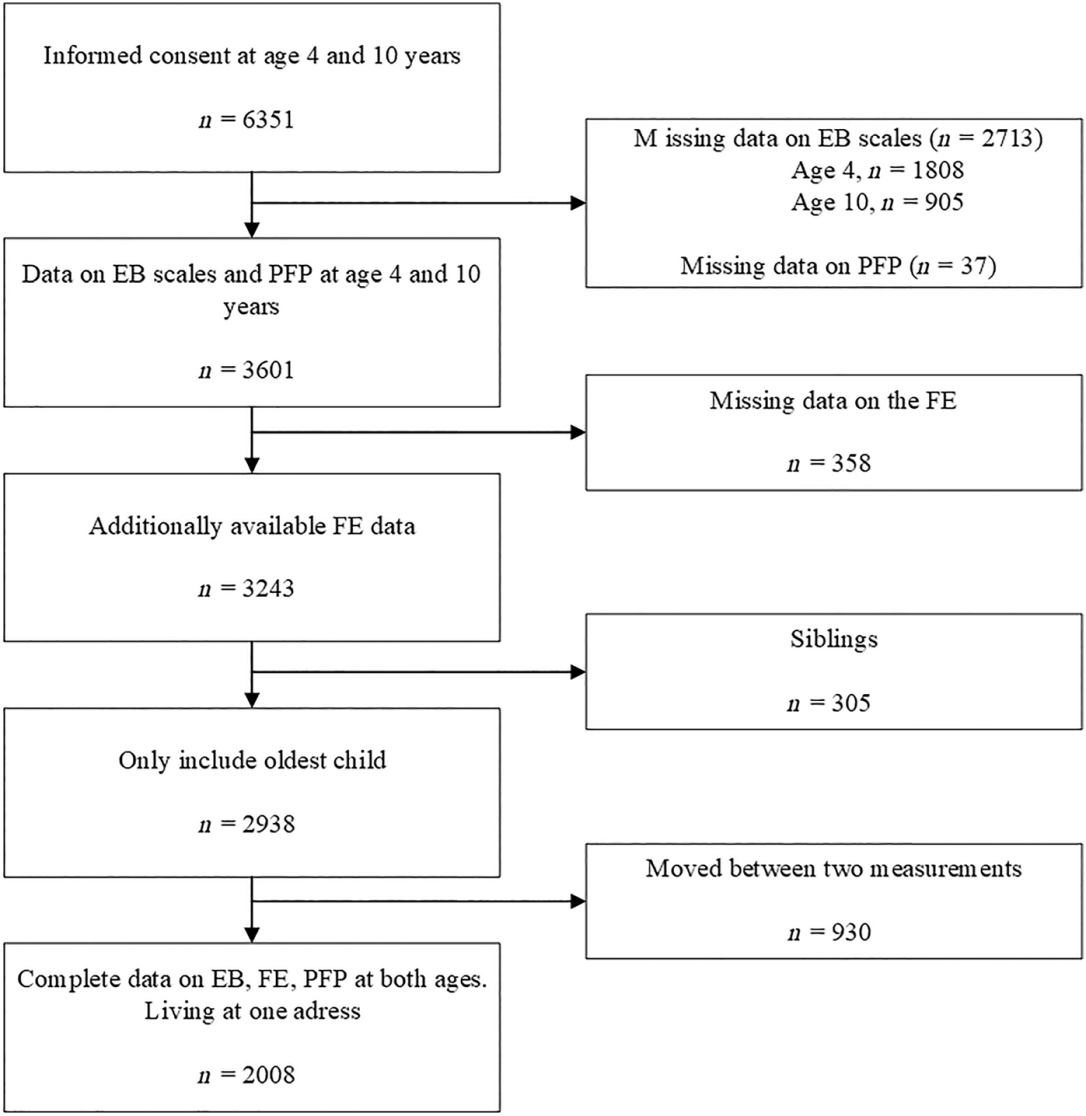
Flowchart exclusion criteria. EB, eating behaviours; FE, food environment; PFP, parental feeding practices.

Of the *n* = 6351 children for which written informed consent was available both at age 4 and age 10, a total of *n* = 3601 individuals (57.3%) had sufficient data on the three eating behaviour scales of interest and parental restriction at both ages (Figure 1). We excluded participants based on missing data on food environment, participants with older siblings who also enrolled in the study, and those who moved addresses between the measurement times to exclude both the effect of clustering within a family and selective migration. Eventually this led to a sample of *n* = 2008 children for the main analysis (Figure 1).

### 2.2 Data‐collection procedure

#### 2.2.1 Independent variable measures: residential food environment

The main exposure of interest was the residential food environment, operationalized as absolute fast‐food exposure, relative fast‐food exposure and a healthiness score, all within a 400‐m radius around the home.

Data on location and type of food outlet, obtained from LOCATUS, have previously been linked with Generation R data.^32^ Using the *x*, *y*‐coordinates of food outlets, data from LOCATUS have been used to map the location of retailers using Geographical Information Systems. All residential addresses of children up to the age of 14 years in Generation R were geocoded. Subsequently, by applying Euclidean buffers of 400 m around the home, the residential food environment was mapped for both time points, mapping food retailers from the year prior to the outcome measure. The 400 m reflects a 5‐min walking distance.^32^ Fast‐food outlets were those outlets labelled as ‘fast‐food outlets’ by LOCATUS, including grillrooms, take‐away outlets and ice cream shops. A previous validation study found good to excellent agreement when validating the dataset using field audit data.^33^

Relative fast‐food exposure has been calculated by dividing the total number of fast‐food outlets by the total number of food outlets in the area. Similarly to Timmermans et al., an average healthiness score, based on the healthiness score of all the food outlets within 400 m of the home, was used to characterize the healthiness of the food environment ranging from −5 (very unhealthy) to +5 (extremely healthy).^34^

#### 2.2.2 Outcome variable measures: children's eating behaviour

Eating behaviours of children have been assessed at age 4–10, between the years 2006 and 2016, using parental questionnaires. At both ages, the questionnaires were comprised of questions from the Child Eating Behaviour Questionnaire (CEBQ).^35^ This multidimensional questionnaire assesses eating styles in children based on the parents' report of the behaviour of the child.^35^, ^36^ As this questionnaire was specifically developed to investigate individual differences in eating styles that might contribute to the development of obesity, it was considered a suitable outcome measure for this study. We have included the subscales food responsiveness (five items) which address the sensitivity to external food cues, enjoyment of food (four items) which assesses the desire to eat, and the combined scale of satiety related behaviour and slowness in eating which consisted out of nine items that were loaded on the same scale.^35^, ^36^ These scales have been validated against behavioural evaluations of food intake.^36^

In general, the CEBQ has shown good internal consistency, as well as test–retest reliability.^35^ All items were assessed on a 5‐point Likert scale ranging from 1 = seldom to 5 = very often. Similarly to previous studies, we calculated a mean item score (MIS) for each subscale for individuals with no more than 25% missing answers per subscale.^17^, ^37^ Reversed item questions have been reversed‐scored. The included subscales showed moderate to high reliability in our main study sample. Cronbach's *α* for satiety responsiveness was 0.81 and 0.85 for age 4 and 10, respectively. Further scores were also considerate good with scores for food responsiveness being 0.84 and 0.86 and for enjoyment of food being 0.89 and 0.87 at age 4 and 10, respectively.

Correlation coefficients reflecting levels of individual stability of the assessed eating behaviour subscales were considered moderate in our sample (ranging from 0.39 to 0.48, *p*‐values all <0.001, Supplementary files). Moderate correlation coefficients reflect room for individual variation in the development of eating behaviours.^17^ Therefore, eating behaviour subscales within our sample are assumed to be susceptible to change over time.

#### 2.2.3 Parental feeding practices at baseline

To assess parental restriction levels at baseline, we used The Child Feeding Questionnaire (CFQ) at age four. The CFQ assesses the child‐feeding perceptions, attitudes, and control practices over children's eating in a seven‐factor model. The model is validated and designed for the use by parents with children between approximately 2 and 11 years old.^38^ For this study we included the subscale parental restriction which assesses to what extent parents limit access to food.^37^, ^38^ The subscale has been measured on a five‐point Likert scale ranging from 1 = never to 5 = always. Similarly to the CEBQ, a MIS has been calculated for individuals with no more than 25% missing items on the restrictiveness scale. The reliability of this subscale was considered moderate with a Cronbach's *α* of 0.60 at age 4.

#### 2.2.4 Baseline characteristics and covariates

Considered covariates included the child's age, ethnicity, sex, net household income and maternal educational level. This information has been extracted from parental questionnaires.

Following the classification of Statistic Netherlands, a child's ethnic background was categorized as Dutch, other‐Western or non‐Western based on the country of birth of the child's parents.^31^ Maternal education level was categorized using the Dutch Standard Classification of Education, distinguishing the following educational levels: high (university degree), mid‐high (bachelor's degree or vocational training), mid‐low (>3 years of secondary school or intermediate vocational training) and low (no education or solely lower vocational training, primary education or ≤3 years of general secondary school).^39^ Net household income was asked for during the measurement wave at 5–10 years old and was classified as high (>€3200/month), intermediate (€2000–€3200/month) and low (≤€2000). We used income measurements at 5 years old as a proxy for the net household income at our first measurement wave.

### 2.3 Data‐analysis procedure

All statistical analyses have been performed in R Studio (version 4.1.2). Two‐sided *p*‐values <0.05 were deemed significant. We described the characteristics of our main sample using means with standard deviations and medians with interquartile ranges for continuous variables. We used percentages for categorical variables. A multiple group summary has been performed whereby the individuals in the main analysis have been compared to individuals that have been excluded at various stages (Supplementary files). Analysis were based complete data, missings have been further summarized in the Supplementary files.

Using individual panel data, whereby individuals (level‐2) are measured at multiple occasions (level‐1) we performed a Random Effect Within Between Model (REWB), using the lmer() function. As described in previous research, a REWB can be considered as an extended version of a fixed‐effects model, allowing level 2 variables to be included. Hereby time‐varying independent variable are decomposed into individual‐specific means (between‐individual estimates) and deviations from the individual‐specific means (within‐individual estimates).^40^, ^41^, ^42^, ^43^

We base ourselves on the simple REWB model, specified by Bell et al., which assumes homogeneous effects across level two entities.^40^ The general model can be described as follows:

$$y_{\mathrm{it}}=\beta_{0}+\beta_{1W}\left(x_{\mathrm{it}}-{\bar{x}}_{i}\right)+\beta_{2B}{\bar{x}}_{i}+\beta_{3}z_{i}+\left(\upsilon_{i}+\varepsilon_{\mathrm{it}}\right),$$

*Y*_*𝑖t*_ represents the time‐varying dependent variable, namely eating behaviour scales, *x*_*𝑖𝑡*_ is a time‐varying independent variable at level 1 whereby ${\bar{x}}_{i}$ represents the individual‐specific mean. The 𝛽_1𝑊_ represents the average within effects of the included time‐varying independent variables, representing how variation in exposure around the individual's mean is related to the outcome. 𝛽_2𝐵_ represents the between‐effects of the time‐varying independent variables, representing how exposure across all participants‐observations relates to the outcome. Subsequently, 𝛽_3_ represents the effect of the time‐invariant variables *z*_*i*_ and thus denotes a between‐subject effect, as a constant variable at the level of the individual cannot have a within subject effect. $\upsilon_{i}$ represent the model's random effects for individual *i*. 𝜖_𝑖𝑡_ represents the model level 1 residuals.

To test whether the association between a changing fast‐food environment and changing eating behaviour scores (i.e. within‐effect) is moderated by levels of parental restriction, we added an interaction with parental restriction at baseline:

$$y_{\mathrm{it}}=\beta_{0}+\beta_{1W}\left(x_{\mathrm{it}}-{\bar{x}}_{i}\right)+\beta_{2B}{\bar{x}}_{i}+\beta_{3}z_{i}+\left(\beta_{4}\left(x_{\mathrm{it}}-{\bar{x}}_{i}\right)*z_{i}\right)+\left(\upsilon_{i}+\varepsilon_{\mathrm{it}}\right),$$

where 𝛽_4_ represents the moderating effect of baseline parental restriction levels on eating behaviour scores in the face of a changing fast‐food environment.

## 3 RESULTS

### 3.1 Sample characteristics

Characteristics of the main sample have been summarized in Table 1. The mean age was 4.0 and 9.7 years during the first and second wave, respectively. In our study sample, most mothers completed a high‐ (35.4%) or a mid‐high education (29.8%). The proportion of parents reporting a net household income >€3200/month increased from 57.3% to 63.0% between the two measurement waves. Approximately half of the children was female (49.8%), and most were classified as native‐Dutch (71.3%).

**TABLE 1:** Descriptive characteristics of the study population.

Variables	Age 4 (*n* = 2008)	Age 10 (*n* = 2008)
Child characteristics		
Girls	1000 (49.8)	
Ethnicity		
Dutch	1432 (71.3)	
Non‐Western	377 (18.8)	
Other‐Western	195 (9.7)	
NA	4 (0.2)	
Age (year)	4.0 ± 0.1^a^	9.7 ± 0.3
Parental characteristics		
Educational level mother		
Low	119 (5.9)^b^	
Mid‐low	496 (24.7)	
Mid‐high	598 (29.8)	
High	710 (35.4)	
NA	85 (4.2)	
Net household income		
<2000 euros/month	216 (10.8)	242 (12.1)
2000–3200	489 (24.4)	398 (19.8)
>3200	1150 (57.3)	1265 (63.0)
NA	153 (7.6)	103 (5.1)
Food environment within 400 m around the home		
Healthiness score	−0.67 ± 1.20	−0.76 ± 1.21
Fast‐food outlets, *n*	1 [0, 3]^c^	1 [0, 3]
Total food outlets, *n*	7 [1, 23]	5 [1, 20]
Relative fast‐food (%)	12.10 ± 16.56	13.78 ± 17.99
Measures of eating behaviour and parental feeding practices		
MIS satiety responsiveness	3.11 ± 0.61	2.57 ± 0.66
MIS enjoyment of food	3.37 ± 0.73	3.61 ± 0.68
MIS food responsiveness	1.60 [1.40, 2.00]	1.60 [1.20, 2.20]
MIS restrictive feeding practices	3.08 ± 0.77	

*Note*: Values are based on observed data (not imputed), frequency of missing data has been summarized in Supplementary files.

Abbreviation: MIS, mean item score; NA, not available.

^a^ Mean ± standard deviation (all such values).

^b^ *n*, (percentage), (all such values).

^c^ Median [interquartile range] (all such values).

Over an average period of 5.7 years' time, the mean healthiness score of the food environment decreased from −0.67 to −0.76, indicating that the food environment became unhealthier over time. The absolute number of fast‐food outlets around children's home remained the same (one outlet) on average. The median of total food outlets decreased over time from seven to five outlets whereby the relative percentage of fast‐food outlets increased from 12.1% to 13.8%. Distributions of changes in the food environment have been summarized in the supplementary materials.

The MIS of satiety responsiveness was significantly higher at age 4 (3.11 points) compared to age 10 (2.57 points) meaning that, on average, the ability to control food intake when feeling full decreased over time. The MIS of enjoyment of food, increased modestly from 3.37 points to 3.61 points, indicating increased general appetite. The median MIS of food responsiveness did not significantly change over time, meaning that on average the eating in response to external food cues did not change.

Comparing our main sample to the individuals that were excluded from the analysis due to missing data, we observed that maternal educational level, net household income and the proportion of children with a Dutch ethnicity were significantly higher in our study sample (Supplementary files). Additionally, the number of fast‐food outlets and the number of total food outlets at baseline were higher in the excluded sample compared to the main sample. Relative fast‐food exposure was comparable to the main sample at baseline. There were no significant differences in the measures of eating behaviour and parental feeding practices at baseline. Comparison per excluded subgroup has been further summarized in the Supplementary files.

When evaluating the role of the changing food environment on eating behaviours within children, we observed that changes in relative fast‐food exposure and healthiness of the food environment were not associated with eating behaviour outcomes. A change in absolute fast‐food exposure, was statistically significant related to a change in satiety responsiveness (Table 2), whereby the effect size was considered marginal. An increase of one fast‐food unit was associated with a 0.02 point (95% confidence interval: 0.00–0.03) increase in MIS of satiety responsiveness. No associations were found between a change in absolute fast‐food exposure and changes enjoyment of food or food responsiveness.

**TABLE 2:** Main effects changes in the food environment and changes in mean item score of eating behaviour subscales.

	Satiety responsiveness		Enjoyment of food		Food responsiveness	
	Estimate (95% CI)	*p*‐Value	Estimate (95% CI)	*p*‐Value	Estimate (95% CI)	*p*‐Value
Within estimates						
Relative fast‐food exposure (+10%‐point)	0.02 (−0.00 to 0.05)	0.05	−0.01 (−0.03 to 0.02)	0.59	−0.02 (−0.05 to 0.01)	0.12
Absolute fast‐food exposure (+1 outlet)	**0.02 (0.00–0.03)**	**0.02**	−0.01 (−0.03 to 0.00)	0.07	−0.01 (−0.03 to 0.00)	0.09
Healthiness score (+ 1 point)	−0.01 (−0.04 to 0.02)	0.62	−0.01 (−0.05 to 0.02)	0.52	0.03 (−0.01 to 0.02)	0.16
Between						
Relative fast‐food exposure (+10%‐point)	−0.00 (−0.02 to 0.01)	0.59	−0.00 (−0.02 to 0.01)	0.80	0.01 (−0.01 to 0.02)	0.38
Absolute fast‐food exposure (+1 outlet)	−0.00 (−0.01 to 0.01)	0.86	0.01 (−0.00 to 0.01)	0.13	−0.00 (−0.01 to 0.01)	0.79
Healthiness score (+ 1 point)	0.02 (−0.01 to 0.04)	0.14	−0.01 (−0.03 to 0.01)	0.44	−0.01 (−0.03 to 0.02)	0.56

*Note*: Estimates are derived from a Random Effect Within Between model adjusted for the time‐varying variables age and income, and the time unvarying variables gender, ethnicity and maternal education level. Measurements were collected at age 4 and 10. Bold values indicate statistical significance at a 95% confidence levels (CI). Regression estimates are based on non‐imputed dataset.

The REWB model did not provide evidence for between‐associations considering all three eating behaviour scales. The fast‐food environment across all participant‐observations, as assessed with the between‐associations, was thus not significantly related to satiety responsiveness, enjoyment of food, or food responsiveness.

The cross‐level interaction terms between parental restriction at baseline with changes the food environment were considered insignificant (*p* > 0.1) (Table 3). In our sample, we thus found no evidence for the moderation of the within‐person associations with parental restriction at baseline.

**TABLE 3:** Associations between changes in the food environment and changes in mean item score of eating behaviour subscales including moderation baseline parental restriction.

	Satiety responsiveness		Enjoyment of food		Food responsiveness	
	Estimate (95% CI)	*p*‐Value	Estimate (95% CI)	*p*‐Value	Estimate (95% CI)	*p*‐Value
Within estimates						
Relative fast‐food exposure (+10%‐point)	0.02 (−0.07 to 0.11)	0.69	0.03 (−0.07 to 0.14)	0.57	0.06 (−0.05 to 0.18)	0.27
Absolute fast‐food exposure (+1 outlet)	0.05 (−0.01 to 0.10)	0.10	−0.03 (−0.10 to 0.03)	0.29	0.04 (−0.03 to 0.11)	0.27
Healthiness score (+ 1 point)	0.04 (−0.09 to 0.018)	0.53	−0.02 (−0.17 to 0.13)	0.80	−0.04 (−0.21 to 0.12)	0.62
Between						
Relative fast‐food exposure (+10%‐point)	−0.00 (−0.02 to 0.01)	0.56	−0.00 (−0.02 to 0.01)	0.80	0.00 (−0.01 to 0.02)	0.57
Absolute fast‐food exposure (+1 outlet)	−0.00 (−0.01 to 0.01)	0.84	0.01 (−0.00 to 0.01)	0.13	−0.00 (−0.01 to 0.01)	0.69
Healthiness score (+ 1 point)	0.02 (−0.00 to 0.04)	0.13	−0.01 (−0.03 to 0.01)	0.44	−0.00 (−0.03 to 0.02)	0.73
Cross‐level interaction parental restriction baseline						
Relative fast‐food exposure (+10%‐point)	0.00 (−0.03 to 0.03)	0.92	−0.01 (−0.04 to 0.02)	0.46	−0.03 (−0.06 to 0.01)	0.12
Absolute fast‐food exposure (+1 outlet)	−0.01 (−0.03 to 0.01)	0.27	0.01 (−0.01 to 0.02)	0.52	−0.02 (−0.04 to 0.01)	0.12
Healthiness score (+ 1 point)	−0.02 (−0.06 to 0.03)	0.44	0.00 (−0.04 to 0.05)	0.91	0.02 (−0.03 to 0.07)	0.39

*Note*: Estimates are derived from a Random Effect Within Between model adjusted for the time‐varying variables age and income, and the time unvarying variables gender, ethnicity and maternal education level. Measurements were collected at age 4 and 10. Bold values indicate statistical significance at a 95% confidence levels (CI). Regression estimates are based on non‐imputed dataset.

## 4 DISCUSSION

In this study, we investigated the impact of changes in the food environment on changes in children's eating behaviours and the potential moderating role of parent's restriction in this association. We contributed to the evidence base by using a longitudinal design and reporting on children's eating behaviours, a more proxime outcome related to childhood overweight and obesity. We observed that more fast‐food outlets around home in an already poor food environment marginally increased the MIS of satiety responsiveness with 0.02 points (within‐effect). A relative increase in fast‐food exposure was not significantly associated with satiety responsiveness. Regardless of how the fast‐food environment was operationalized, no within‐ or between‐effects were found for the sub‐scales enjoyment of food and food responsiveness. Interaction with parental restriction was deemed insignificant.

### 4.1 Strengths and limitations

A considered strength of this study was the inclusion of repeated measurements, whereby temporality adds to the approximation of causality. Additionally, the choice of model is considered a strength as the REWB model, similarly to a fixed‐effects model, produces unbiased estimates of level 1 variables whilst level 2 variables can be included and can be controlled.^40^, ^41^, ^42^, ^43^ This allows us to estimate both the effect of a changing fast‐food environment on changing eating behaviours over time, as well as to estimate the association of fast‐food exposure with eating behaviours across all participant‐observations. In an environment where fast‐food is omnipresent and does not change substantially for the majority of our sample, including a between estimate is of interest. Furthermore, the use of subject‐specific exposures was considered as a strength of this study. As, rather than relying on neighbourhood or census data, we could estimate the effects of the direct living environments of the children. The follow‐up time of approximately 6 years was considered to be sufficient to observe changes both in the food environment as well as in eating behaviour.

A considered limitation was the use of questionnaires to assess the outcomes and moderator. Both the CFQ and the CEBQ are questionnaires that are completed by the child's parents which might induce bias regarding the true eating behaviour of the child and the actual parental feeding styles. However, we expect this did not substantially influence the results as the within estimator accounts for measurement error nested within individuals (level 2 variable).^44^ Our assumptions are strengthened by previous validation of maternal reports of eating behaviour against four aspects of eating behaviour (eating without hunger, caloric compensation, eating rate and energy intake at a meal).^36^ Additionally, it should be considered that the exposure as operationalized in our study was limited to residential food environments at 400 m around the home. This distance was chosen to reflect the area where children and their parents are exposed to shops in the direct vicinity of their homes and allows for comparison with US studies that express characteristics of the environment in quartile‐miles. Future research should aim to include capturing where exposure takes place, including a wider focus on the food environment such as the school environment. Lastly, we excluded children with no information on residential address, and those with more than 25% missing items on eating behaviours subscales. In the Supplementary files (Table S3), we showed the impact of the missing variables on the characteristics of our final sample. Our final sample was not fully representable of the wider underlying population as we excluded lower SES groups more frequently. Although relative fast‐food exposure at baseline was comparable between our study sample and the excluded sample, it should be noted that especially in low‐income areas, fast‐food outlets are more prevalent and increase more rapidly.^6^, ^32^ Apart from the association of unhealthy food environments with lower SES, it has additionally been found that fast‐food consumption differs between education groups and that the co‐occurrence of a poor‐quality resource environment and limited economical resources adds to the inequalities in obesity risk.^6^, ^45^, ^46^, ^47^ Likely, our results are therefore a conservative estimation.

When interpreting our results, we must note that in an environment where the relative abundance of fast‐food was already considerably high, solely small changes in the fast‐food exposure were observed, reflected by the, on average, small increase in relative fast‐food exposure, decrease in healthiness score and stable absolute fast‐food exposure. Not only might this have impacted within estimates, that look at the change in fast‐food exposure related to the change in eating behaviour, but also it might additionally explain the minor and insignificant between‐effects, when the differences between individuals are considered small. The lack of changes in an already ubiquitous fast‐food environment, have previously been mentioned as explanation for lack of findings when it comes to BMI.^32^ Additionally, changes in eating behaviours have been considered small. This is in line with previous research which concluded that eating behaviours are relatively continuous over time, indicated by consistent group ratings.^17^, ^48^, ^49^

The marginal yet significant effect of the absolute fast‐food environment on satiety responsiveness can potentially be caused by the fact that if unhealthy food outlets increase, healthy food sources also tend to increase.^50^ An increase in the absolute number of fast‐food outlets does not inform us on the number of healthy food outlets. If there is a similar or even higher increase in healthy food outlets, the perceived availability of unhealthy food outlets might decrease.^51^ This might explain why there was no significant increase of satiety responsiveness when considering the relative fast‐food exposure measure and healthiness score.

The lack of association between increased relative fast‐food exposure and increased enjoyment of food and food responsiveness is in contrast to our hypothesis that an increase in fast‐food in the residential environment would be associated with increased food approaching behaviours. Arguably the assessed residential food environment does not sufficiently capture the family food environment. Previous research states that, especially at younger ages, the food environment is affecting children mainly through the pathway of shaping parents' purchasing behaviours, food intake and eating behaviours.^19^, ^20^, ^21^, ^22^, ^52^ For further research, it is therefore recommended to use more proximate measures of the family food environment, including which foods are purchased and available within the home. Additionally, direct measures of intake behaviour might be a more suitable alternative when studying the effect of fast‐food exposure on eating behaviours.

This research suggests that in environments with ubiquitous fast‐food outlets, small changes in fast‐food exposure do not seem to have a substantial impact on children's eating behaviours. Considering the high levels of childhood overweight and obesity in many populations across the world, policy action is warranted to avoid further deterioration of the food environment. The absence of evidence should not result in policy inertia. To the contrary, researchers need to advocate for combinations of policies targeting the food environment to curb the levels of overweight and obesity.

Considering the lack of interaction effects for parental feeding practices, it should be noted that the relationship between eating behaviours and restriction is complex. Findings from previous studies indicated that high restriction levels were associated with promoted food responsiveness, eating in the absence of hunger and overweight, supporting the hypothesis that restrictive feeding might disrupt the child's development of self‐control at a later age.^23^, ^24^, ^25^, ^26^ Other studies, however, show signs of bi‐directionality and responsiveness, whereby elevated levels of food responsiveness or overweight move parents to become more restrictive.^27^, ^28^, ^29^ Based on the results of our study, parental restriction does not seem to play a role in the relationship between residential fast‐food exposure and eating behaviours in children.

## 5 CONCLUSION

This study did not identify substantial effects of the residential fast‐food environment on eating behaviours associated with childhood overweight and obesity. We therefore support further research looking at more proximate outcomes including dietary intake and purchasing behaviour.

## AUTHOR CONTRIBUTIONS

TAM, MAB and FJMM conceptualized the study. FJMM contributed to the data set‐up in an earlier research project. TAM conducted the literature search, conducted the analysis, drafted the initial manuscript and revised the final manuscript. MAB, FJMM, PWJ, JOG and FJvL thoroughly reviewed all stages of the manuscript. All authors were responsible for reading and approving the final manuscript.

## FUNDING INFORMATION

The Generation R Study was made possible by financial support from the Erasmus Medical Centre, Erasmus University Rotterdam and The Netherlands Organisation for Health Research and Development, The Netherlands Organisation for Scientific Research, the Ministry of Health, Welfare and Sport and the Ministry of Youth and Families. This project has received funding from the Erasmus Initiative ‘Smarter Choices for Better Health’.

## CONFLICT OF INTEREST STATEMENT

The authors declare that they have no competing financial interests.

## Supporting information


**Data S1.** Supporting information.

## Data Availability

The data that support the findings of this study are available from Generation R, but restrictions apply to the availability of these data, which were used under licence for the current study, and so are not publicly available. Data are however available from the authors upon reasonable request and with permission of Generation R.
