# Supplementary material for: Effect of fast‐food environments on children's eating behaviour: A random effect within between analysis within the Generation R Study
Source: Pediatr Obes. 2024 Sep 29;20(4):e13175. doi: 10.1111/ijpo.13175 (PMC11936701; doi:10.1111/ijpo.13175)
Supplement: Supplementary file 1 — Data S1. Supporting information. [file IJPO-20-e13175-s001.pdf]

Effect of Fast-food Environments on Children's Eating Behaviour: A Random Effect Within Between Analysis within the Generation R Study

Thera A.M. Peeters[1,2], Famke J. M. Mölenberg[1], Pauline W. Jansen[2,3,4], Joost Oude Groeniger[1,5], Frank J. van Lenthe[1,6], Mariëlle A. Beenackers[1]

Affiliations

1. Department of Public Health, Erasmus MC, Rotterdam, The Netherlands.
2. The Generation R Study Group, Erasmus MC, Rotterdam, The Netherlands.
3. Department of Child and Adolescent Psychiatry/Psychology, Erasmus MC, Rotterdam, The Netherlands
4. Department of Psychology, Education and Child Studies, Erasmus University, Rotterdam, The Netherlands.
5. Department of Public Administration and Sociology, Erasmus University, Rotterdam, the Netherlands
6. Department of Human Geography and Spatial Planning, Utrecht University, Utrecht, the Netherlands

Corresponding author

Thera A.M. Peeters, MSc, Department of Public Health, Erasmus MC, University Medical Centre Rotterdam, P.O. Box 2040, 3000 CA Rotterdam, The Netherlands. E-mail: t.peeters@erasmusmc.nl

Other authors

Famke J. M. Mölenberg, Dr, Department of Public Health, Erasmus MC, University Medical Center Rotterdam, P.O. Box 2040, 3000 CA Rotterdam, The Netherlands. E-mail: f.molenberg@erasmusmc.nl

Pauline W. Jansen, prof. Dr, The Generation R Study Group, Erasmus MC, University Medical Center Rotterdam, P.O. Box 2040, 3000 CA Rotterdam, The Netherlands. E-mail: p.w.jansen@erasmusmc.nl

Joost Oude Groeniger, Dr, Department of Public Health, Erasmus MC, University Medical Center Rotterdam, P.O. Box 2040, 3000 CA Rotterdam, The Netherlands. E-mail: j.oudegroeniger@erasmusmc.nl

Frank J. van Lenthe, prof. Dr, Department of Public Health, Erasmus MC, University Medical Center Rotterdam, P.O. Box 2040, 3000 CA Rotterdam, The Netherlands. E-mail: f.vanlenthe@erasmusmc.nl

Mariëlle A. Beenackers, Dr, Department of Public Health, Erasmus MC, University Medical Center Rotterdam, P.O. Box 2040, 3000 CA Rotterdam, The Netherlands. E-mail: m.beenackers@erasmusmc.nl

Contents

Appendix 1 – Change in the food environment over time

Appendix 2 – Change in eating behavior over time

Appendix 3 - Continuity and stability of eating behaviours and parental restriction

Appendix 4 - Comparison main sample and excluded samples

Appendix 5 – Missing data

Appendix 1 - Change in the food environment over time

Histograms are displaying change in the food environment over a time period of 5.7 years, from baseline (4 years old) to follow up (10 years old).

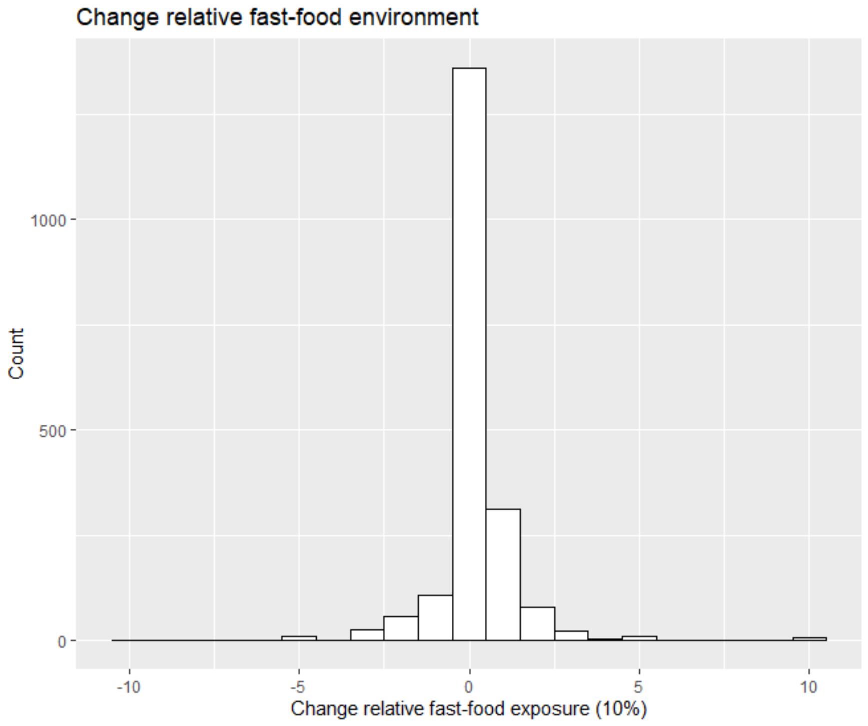

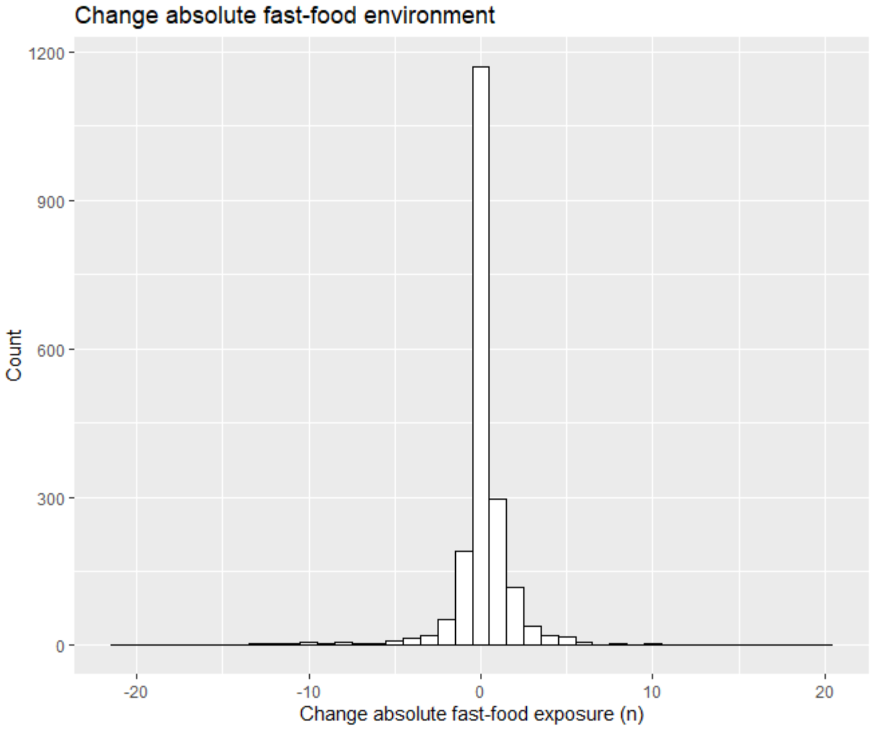

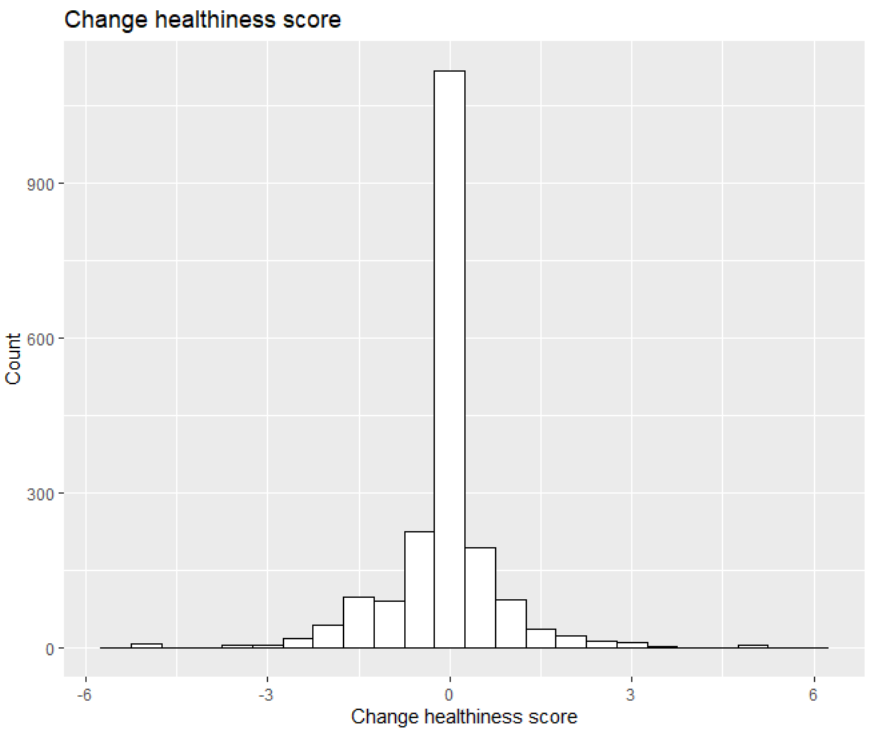
Change healthiness score
Count
900
600
300
0
-6
-3
0
3
6
Change healthiness score

Appendix 2 - Change in the eating behaviour over time

Histograms are displaying change in eating behaviour subscales over a time period of 5.7 years, from baseline (4 years old) to follow up (10 years old).

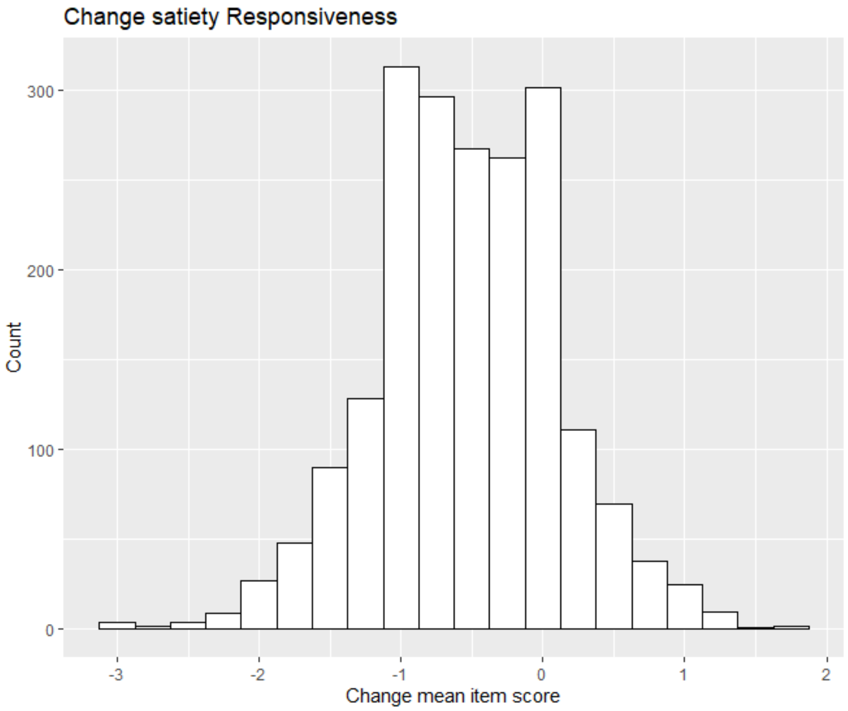

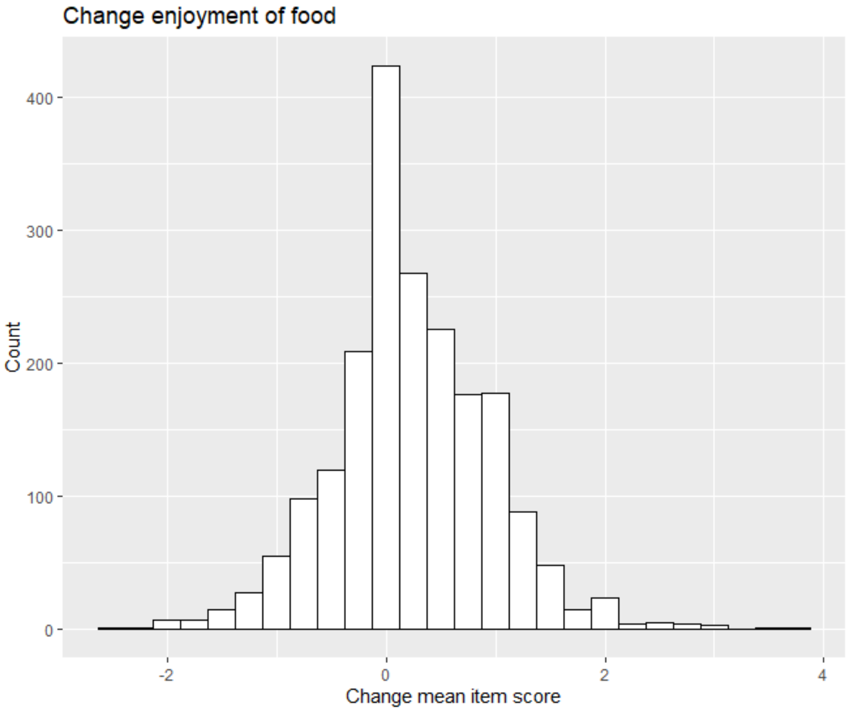

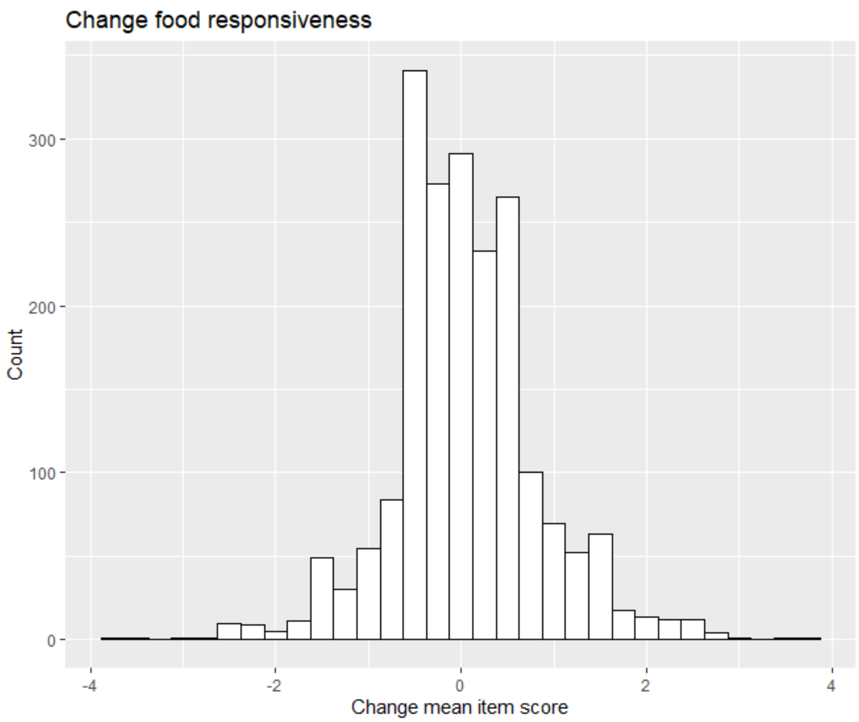
Change food responsiveness
Count
300
200
100
0
-4
-2
0
2
4
Change mean item score

Appendix 3 - Continuity and stability of eating behaviours and parental restriction

Measures of individual stability and continuity are given in table S1 and S2. Continuity, given by mean group level change, reflects consistency of group ratings over time. Stability, given by correlation coefficients, reflects consistency of individual ratings over time. As indicated by Derks et al. (2019), moderate to low correlations suggest there is room for potential individual variation in eating behaviours over time.

Table S1: Mean group level change between 4 and 10 years old

Questionnaire Item	Age 4	Age 10	Change (95% CI)	Significance of mean group level change
**Satiety responsiveness**	3.11 ± 0.61[a]	2.57 ± 0.66	-0.54 (-0.57;-0.51)	**p<0.001**[c]
**Enjoyment of food**	3.37 ± 0.73	3.61 ± 0.68	0.24 (0.20;0.26)	**p<0.001**[c]
**Food responsiveness**	1.60[1.40, 2.00][b]	1.60[1.20, 2.20]	0.00 (-0.10; 0.00)	p=0.09[d]

a: mean ± standard deviation (SD) (all such values)
b: median [IQR]
c: values have been based on paired t-test
d: As the MIS of food responsiveness were not normally distributed, change has been assessed with a Wilcoxson signed ranks test.
Note: bold text indicates statistically significant finding at a 95% confidence level.

Table S2: Correlation coefficients of MISs of eating behavioural subscales, indicating individual stability between 4 and 10 years old

CEBQ subscales	R	p-value
**Satiety responsiveness**	0.48	< **0.001**
**Enjoyment of food**	0.47	< **0.001**
**Food responsiveness**	0.39	< **0.001**

Note: bold text indicates statistically significant finding at a 95% confidence level.

Appendix 4 - Comparison main sample and excluded samples

In addition to the comparisons as described in the main article between the main sample and the excluded samples, the following observations have been made. The proportions of mothers with a high educational level and net household income level >3200 were significantly higher in our study sample, compared to the excluded sample (n=4343, **Table S3**). We observed the same when comparing the main sample solely to individuals that were excluded due to missing information on eating behaviour at age ten (lost to follow-up, n=905; **Table S3**). Individuals excluded due to moving house (moved house, n=930) did not show significant differences in educational level, however, they did show significant differences in parental characteristics when it came to net household income. Again, the proportion net-household income >3200 was higher in our sample compared to children that moved (**Table S3**).

Considering child characteristics, the excluded sample significantly differed from the main sample concerning ethnicity. Notably, the proportion of children with a non-western background was much higher in the excluded sample compared to the main sample (34.2% compared to 18.8%). The proportion of non-western children was also significantly higher in children that were lost to follow up (**Table S3**). Mean age was also considered significantly different between the main sample and the excluded sample and lost to follow up sample, however, this was not considered of noteworthy importance. The sample of moved individuals did not differ significantly from the main sample when it came to child characteristics (**Table S3**).

The difference in (fast-) food outlets was considered significantly different between the excluded samples and our study sample. Both the number fast-food outlets and number of total food outlets were higher than in the main sample. Relative fast-food exposure and healthiness score were comparable across samples. There was no significant difference in the measures of eating behaviour and parental feeding practices across the samples (**Table S3).**

Table S3: Comparison of descriptive characteristics between main sample, excluded sample, lost to follow up sample, and moved sample at baseline.

	**Main Sample (n=2008)**	**Excluded sample (n= 4343)**	**p-value**	**Lost to follow-up sample (n= 905)**	**p-value**	**Moved sample (n=930)**	**p-value**
**Parental characteristics**							
Educational level mother			**<0.001***		**<0.001**		**0.02**
Low	119 (5.9) [a]	394 (9.1)		109 (12.0)		47 (5.1)	
Mid-Low	496 (24.7)	939 (21.6)		190 (21.0)		221 (23.8)	
Mid-High	598 (29.8)	874 (20.1)		188 (20.8)		270 (29.0)	
High	710 (35.4)	967 (22.3)		220 (24.3)		325 (34.9)	
Net household income			**<0.001**		**<0.001**		**<0.001**
≤2000	216 (10.8)	737 (17.0)		148 (16.4)		157 (16.9)	
2000-3200	489 (24.4)	723 (16.6)		152 (16.8)		190 (20.4)	
>3200	1150 (57.3)	1511 (34.8)		351 (38.8)		475 (51.1)	
**Child characteristics**							
Sex			0.84		0.28		0.41
boy	1008 (50.2)	2162 (49.8)		474 (52.4)		451 (48.5)	
girl	1000 (49.8)	2180 (50.2)		431 (47.6)		479 (51.5)	
Ethnicity			**<0.001**		**<0.001**		0.11
Dutch	1432 (71.3)	2315 (53.3)		515 (56.9)		627 (67.4)	
Non-Western	377 (18.8)	1484 (34.2)		296 (32.7)		204 (21.9)	
Other-Western	195 (9.7)	355 (8.2)		76 (8.4)		95 (10.2)	
Age	4.0 ± 0.1[b]	4.1 ± 0.1	**<0.001**	4.1 ± 0.1	**<0.001**	4.1 ± 0.1	0.47
**Food environment within 400m around the home**							
Healthiness score	-0.67 ± 1.20	-0.72 ± 1.07	0.19	-0.69 ± 1.04	0.79	-0.76 ± 1.07	0.05
Fast food outlets, n	1.00 [0.00, 3.00][c]	2.00 [0.00, 4.25]	**<0.001**	1.00 [0.00, 4.00]	**<0.001**	2.00 [0.00, 5.00]	**<0.001**
Total food outlets, n	7.00 [1.00, 23.00]	11.00 [1.00, 33.25]	**<0.001**	11.00 [1.00, 31.00]	**<0.001**	13.00 [2.00, 38.75]	**<0.001**
Relative fast food (%)	12.10 ± 16.56	12.02 ± 14.29	0.86	11.99 ± 13.99	0.86	12.44 ± 14.06	0.59
**Measures of eating behaviour and parental feeding practices**							
MIS satiety responsiveness	3.11 ± 0.61	3.09 ± 0.64	0.23	3.06 ± 0.64	**0.026**	3.13 ± 0.64	0.42
MIS enjoyment of food	3.37 ± 0.73	3.37 ± 0.74	0.91	3.36 ± 0.79	0.767	3.39 ± 0.71	0.64
MIS food responsiveness	1.60 [1.40, 2.00]	1.60 [1.20, 2.05]	0.27	1.60 [1.20, 2.20]	0.917	1.60 [1.20, 2.00]	0.16
MIS restriction	3.08 ± 0.77	3.07 ± 0.79	0.66	3.09 ± 0.81	0.654	3.10 ± 0.77	0.41

a: n, (percentage), (all such values)
b: mean ± standard deviation (SD) (all such values)

c: median [IQR] (all such values)
MIS: Mean item score

Appendix 5- Missing data

Table S4: Proportion missing in main sample, excluded sample, lost to follow up sample and moved sample at baseline.

	Main sample (n=2008)	Excluded sample (n=4343)	Lost-to-follow up sample (n=905)	Moved sample (n=930)
**Parental characteristics**				
Educational level mother	85 (4.2) [a]	1169 (26.9)	198 (21.9)	67 (7.2)
Net household income	153 (7.6)	1372 (31.6)	254 (28.1)	108 (11.6)
**Child characteristics**				
Sex		1 (0.0)		
Ethnicity	4 (0.2)	189 (4.4)	18 (2.0)	4 (0.4)
Age		1732 (40)		
**Food environment within 400m around the home**		2195 (51)	94 (10)	
**Measures of eating behaviour and parental feeding practices**				
MIS satiety responsiveness		1764 (41)		
MIS enjoyment of food		1795 (41)		
MIS food responsiveness		1763 (41)		
MIS restriction		1807 (42)	10 (1.1)	

a: n, (percentage), (all such values)
